# Probing Electronic Band Structure of Monolayer MoS_2_ in Gate-Controlled Resonant Tunneling Diodes

**DOI:** 10.1021/acsami.4c21712

**Published:** 2025-04-15

**Authors:** Chengjie Zhou, Hui Li, Zhenqiao Huang, Chun Yu Wan, Zijing Jin, Junwei Liu, Jiannong Wang

**Affiliations:** † Department of Physics, The Hong Kong University of Science and Technology, Hong Kong, China; ‡ National Key Laboratory of Optoelectronic Information Acquisition and Protection Technology and Institute of Physical Science and Information Technology, Anhui University, Hefei 230601, China

**Keywords:** TMDCs, Single-barrier Resonant Tunneling, Electronic Band Structure, Density of States, van der Waals Heterostructure

## Abstract

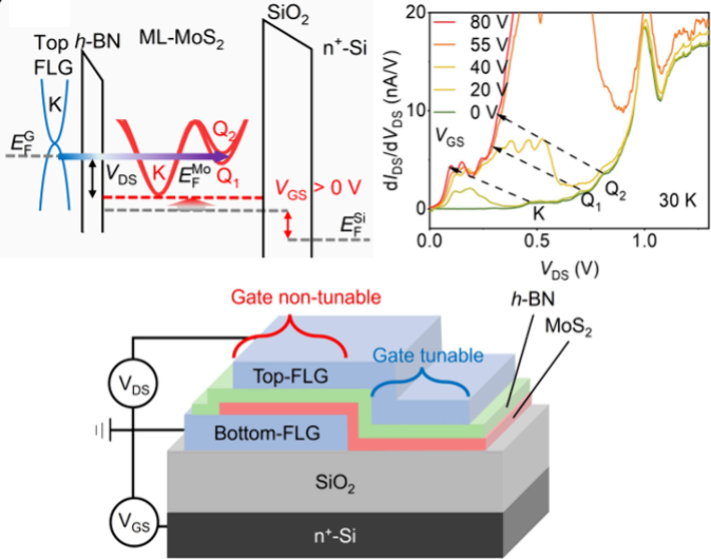

Experimental determination of band structures of monolayer transition metal dichalcogenides (TMDCs) is crucially important in the design and tailoring of the properties of TMDCs. Resonant tunneling spectroscopy (RTS) is an effective technique to probe the band structures of low-dimensional systems by measuring the density of states (DOS) and energy dispersions. Here, we report the investigation of the band structure of monolayer MoS_2_ (ML-MoS_2_) in a gate-controlled resonant tunneling diode. Three distinct resonant tunneling kinks are observed in the characteristic current–voltage curves at 0.47, 0.70, and 0.81 V, respectively, which correspond to the conduction band local minimum of ML-MoS_2_ at K, Q_1_, and Q_2_ points. When applying a large positive gate voltage to enhance ML-MoS_2_ conductivity, the three resonant kinks shift to lower bias at 0.10, 0.32, and 0.39 V, respectively, which is in excellent agreement with the theoretical calculations. Our work offers an effective and more precise way to explore the electronic band structures of TMDCs using RTS.

## Introduction

Mapping the electronic band structures of monolayer transition metal dichalcogenides (TMDCs) through electrical transport measurements offers a straightforward way to explore the underlying mesoscopic physics behind their fascinating physical properties, which has attracted substantial research interest.^1−14^ Resonant tunneling spectroscopy (RTS) has provided an easily accessible route to probe the energy dispersion of electronic band structures. The RTS technique was first demonstrated in the double-barrier quantum well structures of conventional semiconductor heterostructures.^15−19^ Later, it extended to the two-dimensional van der Waals heterostructures with great success, especially in the metal/*h*-BN/metal, metal/*h*-BN/graphene, and graphene/*h*-BN/graphene systems.^20−31^ More recently, it has been applied to investigate phonons or defects in *h*-BN/graphene^32^ and TMDCs,^33,34^ magnons in some ferromagnets,^35^ the moiré bands in twisted bilayer graphene,^36,37^ and the localized conduction bands or valence bands around K or Γ points in TMDCs.^7,38−41^

In principle, RTS measures the resonant tunneling conductance of charge carriers across the heterostructures when the Fermi level of the probe material aligns with the energy band of the target material. The requirements of momentum and energy conservation in the resonant tunneling process allow it to reveal the electronic band structures. However, the insulating feature of the monolayer semiconductor TMDCs introduces the relatively large contact resistance in the RTS measurements,^7,38^ such as monolayer MoS_2_ (ML-MoS_2_), which limits the quantitative accuracy of the measurements significantly. The accurate measurement of the electronic band structures of monolayer TMDCs using the RTS remains a demanding challenge.

Here we fabricated a gate-controllable resonant tunneling diode (RTD) to achieve an accurate measurement of the conduction band structure of ML-MoS_2_ using few-layer graphene (FLG) as a probe. In the current–voltage, i.e., *I*–*V,* curve or RTS of the RTD, the features corresponding to the energy band extremes at K, Q_1_, and Q_2_ points in the first Brillouin zone of the conduction band of ML-MoS_2_ are clearly observed. The energies deduced from these features are in reasonable agreement with the results calculated by density functional theory (DFT). By applying a gate voltage to increase the conductance of ML-MoS_2_ so as to reduce the contact resistance, the accuracy of deduced energies from RTS is greatly improved and an excellent agreement with the DFT calculated results has been achieved. Our findings establish an easily accessible and accurate way for probing the electronic band structures of monolayer semiconducting TMDCs using RTS.

## Results and Discussion

**Figure 1:**
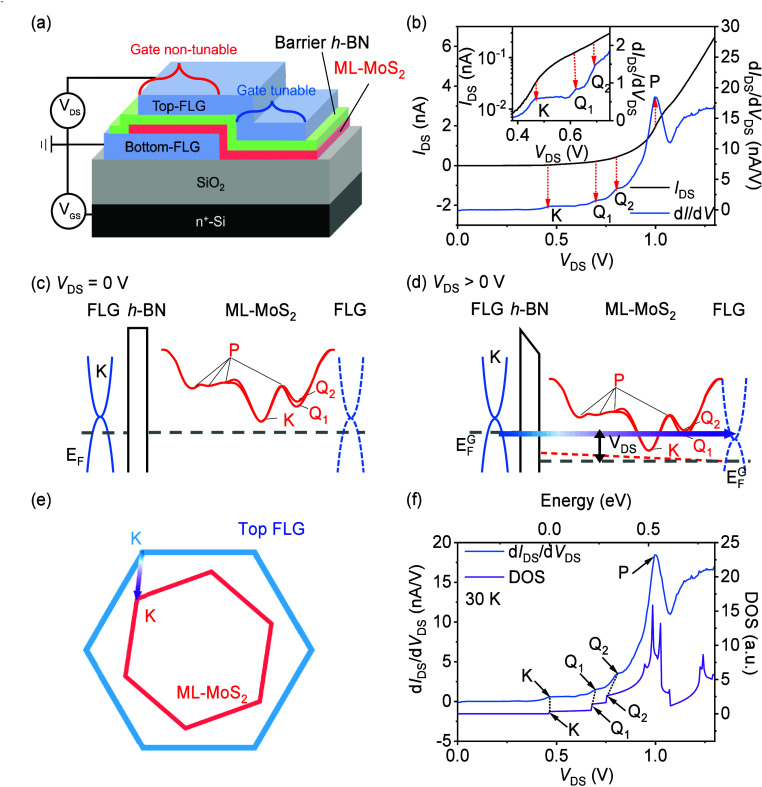
(a) Schematic structure of a single-barrier resonant tunneling diode (top-FLG/*h*-BN/ML-MoS_2_/bottom-FLG) on a SiO_2_/n^+^-Si substrate. The region on the bottom graphite indicated by the red bracket is gate non-tunable, and the region not on the bottom graphite indicated by the blue bracket is gate tunable. (b) The measured *I*_DS_–*V*_DS_ curve (black curve) and d*I*/d*V*–*V*_DS_ curve (blue curve) at 30 K and *V*_GS_ = 0 V. The four resonant kink/peaks are indicated by red dashed arrows and labeled as K, Q_1_, Q_2_, and P points, respectively. Inset is an *I*_DS_–*V*_DS_ curve in a semilogarithmic scale and d*I*/d*V*–*V*_DS_ curve (blue curve) at an enlarged region; three kinks are labeled as K, Q_1_, and Q_2_. (c, d) Schematic band alignment of a gate non-tunable region under *V*_DS_ = 0 (c) and *V*_DS_ > 0 (d), where the bias-induced quasi-Fermi level shift in FLG is ignored for simplicity. (e) Schematic alignment of the first Brillouin zone in an RTD, where the blue and red hexagons correspond to the first Brillouin zone of top-FLG and ML-MoS_2_, respectively. Note the top-FLG and ML-MoS_2_ are randomly aligned. (f) The measured d*I*/d*V*–*V*_DS_ curve (blue curve) and the calculated DOS–energy curve (purple curve) of the ML-MoS_2_ conduction band. The K point of the calculated curve is aligned to the first kink of the experimental curve intentionally.

Figure 1(a) schematically shows the single-barrier RTD structure. The single barrier RTD is a four-layer van der Waals heterostructure (top-FLG/*h*-BN/ML-MoS_2_/bottom-FLG). The top FLG (3–5 nm) serves as the probe or carrier injector or source electrode, the thin *h*-BN (2–3 nm) serves as the tunneling barrier, the ML-MoS_2_ serves as the target material, and the bottom-FLG (3–5 nm) is used to achieve better electrical contact with ML-MoS_2_.^3,7,42,43^ The single-barrier RTD was fabricated on an n^+^-Si substrate capped with 300 nm SiO_2_ by the standard mechanical exfoliation and pick-up method,^44,45^ as detailed in the Experimental Section. The optical image of an RTD is shown in Figure S1 of Supporting Information Note 1. Since FLG is a good conductor, it will shield the ML-MoS_2_ from the influence of the gate electric field. Therefore, only the ML-MoS_2_ that is not in contact with the bottom FLG could be gate tunable. Thus, our device could be categorized into two separated regions: one is immune to the gate voltage, i.e., the gate non-tunable region, as indicated by the red bracket in Figure 1(a), and the other region indicated by the blue bracket is the gate tunable region. The validity of the categorization of two regions has been discussed in Figure S2 of Supporting Information Note 2.

We first measured *I*_DS_–*V*_DS_ characteristics at zero gate voltage (*V*_GS_ = 0 V). When there is no gate voltage applied, MoS_2_ on SiO_2_ is normally nonconducting.^46,47^ The measured tunneling process mainly occurs at the gate non-tunable region from the top-FLG to bottom-FLG directly, where the insulating MoS_2_ and *h*-BN together work as a barrier. Figure 1(b) plots the characteristic *I*_DS_–*V*_DS_ and d*I*/d*V*–*V*_DS_ curves of a single-barrier RTD measured at *T* = 30 K and *V*_GS_ = 0 V. Four kink/peaks, labeled as K, Q_1_, Q_2_, and P, are observed, where positive *V*_DS_ is defined as electrons are injected from the top FLG to ML-MoS_2_. The four kink/peaks locate at about 0.47, 0.70, 0.81, and 1.00 V, respectively, as indicated by red dashed arrows. Similar features are also observed in other devices, as detailed in Figure S3 of Supporting Information Note 3.

To understand the origin of these distinct features, we plot the schematic band alignments of the single barrier RTD at *V*_DS_ = 0 V and *V*_DS_ > 0 V, as shown in Figures 1(c) and 1(d), respectively. The schematic energy bands of top FLG are drawn in blue and the conduction bands of ML-MoS_2_ are drawn in red where the sharp increases of DOS at K, Q_1_, and Q_2_ are indicated, and P includes all local maxima near 0.5 eV above K, as detailed in Figure S4 of Supporting Information Note 4. At *V*_DS_ = 0 V, the equilibrium Fermi level (*E*_*F*_) of the RTD (dashed line) is lower than the conduction band minima at the K point of ML-MoS_2_,^7,8,48,49^ suggesting that electrons in FLG cannot tunnel to ML-MoS_2_. When the single barrier RTD is positively biased, i.e., *V*_DS_ > 0 V, the quasi-Fermi level of FLG (*E*_*F*_^*G*^) will move upward relative to the quasi-Fermi level of ML-MoS_2_ (*E*_*F*_^*Mo*^). As *V*_DS_ increases, it would align with the sharp increases of DOS at K, Q_1_, and Q_2_ points and local maxima represented by P in the conduction band of ML-MoS_2_ in succession, as illustrated in Figure 1(d). It is noted that such alignment in energy cannot lead to the occurrence of direct momentum-conserved tunneling between FLG and ML-MoS_2_ as the K point in the reciprocal space of FLG is far away from that of ML-MoS_2_ due to the large lattice mismatch between FLG and ML-MoS_2_, as illustrated in Figure 1(e). However, the momentum conservation in resonant tunneling could be satisfied by the assistance of phonon emissions/absorptions.^7,22,32^ This phonon-assisted resonant tunneling leads to the observed features at 0.47, 0.70, 0.81, and 1.00 V in *I*_DS_–*V*_DS_ and d*I*/d*V*–*V*_DS_ curves of the single barrier RTD as the quasi-Fermi level of top-FLG is approaching the sharp increases and local maxima of DOS in the conduction band of ML-MoS_2_ at the K, Q_1_, Q_2_, and P points, respectively.

The above qualitative explanation is further verified by the comparison with the theoretical calculation of the energy dependence of DOS of ML-MoS_2_ (see the purple curve in Figure 1(f)). Three sharp increases identified in the purple curve correspond to local maxima of the DOS in the conduction band of ML-MoS_2_ at the K, Q_1_, and Q_2_ points, respectively. The K point of the calculated curve is aligned to the first kink of the experimental curve intentionally with the assumption of the conduction band minima located at *V*_DS_ = 0.45 V. These sharp increases are consistent with our experimental observations of three kinks arising from the tunneling between the top-FLG and ML-MoS_2_ (see purple curve in Figure 1(f)). And the peak P comes from the local maxima of DOS around 0.5 eV above K. However, the energy difference between the K and Q_2_ points is measured to be ∼0.34 eV, which is larger than the theoretical calculation of ∼0.29 eV.^50,51^ Such deviation is believed to originate from the ML-MoS_2_ with insulating characteristics at low temperatures.^46^ In this gate non-tunable region, the insulating MoS_2_ serving as a part of tunneling barrier will exhibit band bending when a positive bias is applied, as indicated by the inclined red dashed line in Figure 1(d). The larger the bias between the top- and bottom-FLG, the more pronounced band bending forms in the MoS_2_/*h*-BN layer, which leads to a broadening of K–Q splitting. Nevertheless, the overall information on the band structures in the first Brillouin zone of ML-MoS_2_ has been effectively mapped out through the phonon-assisted resonant tunneling in our single-barrier RTD.

**Figure 2:**
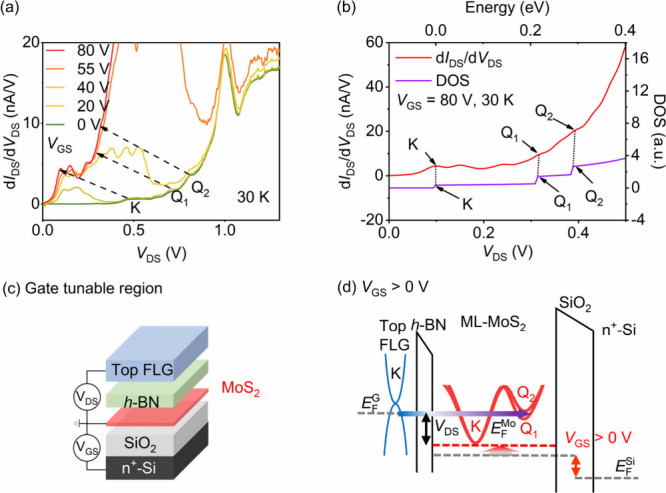
(a) d*I*/d*V*–*V*_DS_ curves at gate voltages indicated from 0 to 80 V. The shifts of the three resonant kinks K, Q_1_, and Q_2_ are indicated by the black dashed arrows. (b) Comparison between the calculated DOS–energy curve (purple curve) and the measured d*I*/d*V*–*V*_DS_ curve (red curve) of ML-MoS_2_ at a gate voltage of 80 V. The K point of the calculated curve is aligned to the first kink of the experimental curve intentionally. (c) Schematic structure of the gate tunable region. (d) Schematic band alignment of a single-barrier RTD with the *V*_GS_ being sufficiently large to tune MoS_2_ into conducting in the gate tunable region.

In the gate tunable region, the insulating characteristics of ML-MoS_2_ can be reduced to improve the mapping accuracy of the band structures by introducing gate voltages. At a gate tunable region as schematically shown in Figure 2(c), applying the positive gate voltage to the n^+^-Si substrate and grounding the ML-MoS_2_ will shift its quasi-Fermi level toward the conduction band, which enhances the conductivity of ML-MoS_2_ and reduces the contact resistance of MoS_2_/Bottom-FLG. This is clearly evidenced in the measured gated d*I*/d*V*–*V*_DS_ curves in Figure 2(a), and the three kinks shift to lower bias gradually when the positive gate voltage increased from 0 to 80 V, as indicated by the black dashed arrows. At *V*_GS_ = 80 V, the kinks corresponding to K, Q_1_, and Q_2_ points are shifted to 0.10, 0.32, and 0.39 V, respectively. It is noted that the K point still locates at positive bias larger than 0 V, which indicates the quasi-Fermi level of MoS_2_ is still below the conduction band edge and suggests the enhanced conductivity may mainly come from the metallic edge of MoS_2_.^52−57^ However, this shift effect induced by positive gate voltage diminishes and is eventually eliminated at a larger positive *V*_DS_ range. As shown in Figure 2(a), all the measured d*I*/d*V*–*V*_DS_ curves converge at *V*_DS_ > 1.2 V. This phenomenon is related to the structural configuration of our single-barrier RTD. When applying *V*_DS_ and *V*_GS_ simultaneously, the RTD can be regarded as a dual-plate capacitor that determines the charging status of ML-MoS_2_, as detailed in equation S1 of Supporting Information Note 5. Such a dual-plate capacitor effect could be also evidenced from the RTS spectrum at some specific conditions with a net charge close to 0. As shown in Figure 2(a), the RTS spectrum becomes noisy accompanied with some multiple peaks between 0.4 and 0.55 V under *V*_GS_ = 40 V, which is believed to be related to the almost zero net charge in ML-MoS_2_.

At a gate voltage of 80 V, the energy difference between the K and Q_2_ points is measured to be ∼0.29 eV (red curve in Figure 2(b)). We note there are multiple newly appearing peaks between the shifted K and Q_1_, which are believed to be related to the defect or phonon states in MoS_2_ or barrier *h*-BN.^32−34^ The elimination and identification of the defect and phonon states could be examined by using higher quality crystals^58,59^ or the detailed gate-dependent measurements,^32^ which may be a promising subject for future study. This energy difference (∼0.29 eV) is smaller than that measured at zero gate voltage (∼0.34 eV), resulting in an excellent agreement with the theoretical calculation of 0.29 eV, as shown in Figure 2(b), where the K point of the calculated curve is aligned to the first kink of the experimental curve at 0.10 V intentionally. Such an improvement in the measurement accuracy is due to the suppressed insulating characteristics of ML-MoS_2_ under positive gate voltages, as schematically illustrated in Figure 2(d). When applying the positive gate voltages, the quasi-Fermi level of ML-MoS_2_ moves upward gradually, as indicated by the wide gray to red arrow in Figure 2(d). This makes the ML-MoS_2_ more conductive and leads to the dominant tunneling process to occur in the gate tunable region between the top-FLG and MoS_2_, as indicated by the horizontal red dashed line in Figure 2(d). In comparison with the zero gate voltage case, a smaller K–Q splitting is expected, which enables a more accurate probing of the energy separation between different conduction band minima of ML-MoS_2_.

**Figure 3:**
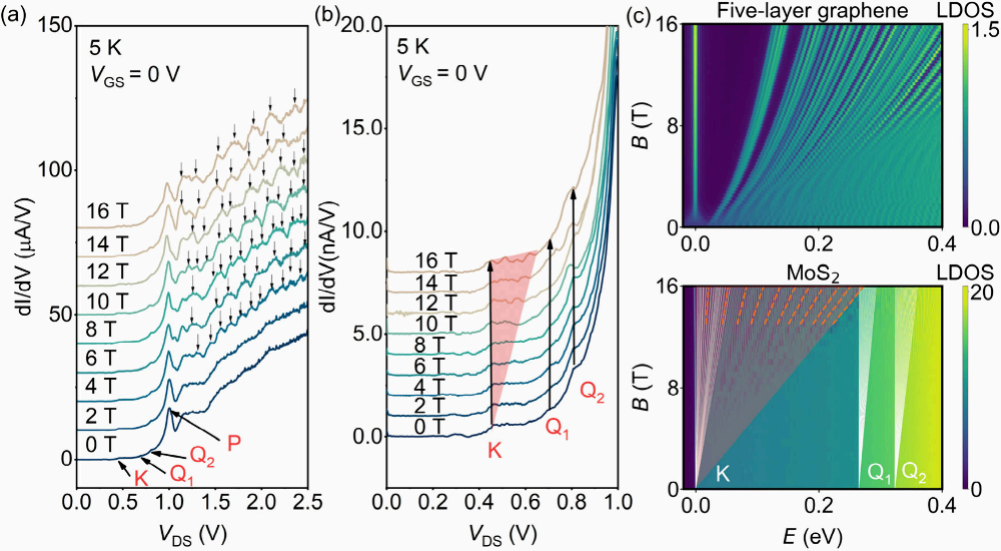
(a) Measured d*I*/d*V* curves under different ***B***-fields applied parallel to the tunneling current from 0 to 16 T (curves are offset for clarity); the ***B***-field step is 2 T. The black arrows indicate the Landau levels of FLG. (b) Zoom-in view of d*I*/d*V*–*V*_DS_ curves under different ***B***-fields of (a). The kink at the K point gradually broadened (red shadow region), and the kink width of Q_1_ and Q_2_ is almost unchanged with increasing ***B***-fields for ML-MoS_2_. (c) Tight-binding model simulated Landau fan diagram of a five-layer graphene (upper panel) and an ML-MoS_2_ (lower panel) under out-of-plane ***B***-fields from 0 to 16 T. Landau levels in ML-MoS_2_ of different valleys of K and Q_1_ and Q_2_ are indicated by white lines.

Figure 3(a) shows the measured d*I*/d*V*–*V*_DS_ curves under external magnetic fields (***B***-fields) applied parallel to the tunneling current at 5 K and *V*_GS_ = 0 V. At zero ***B***-field, the positions of the kinks/peaks are the same as that observed at 30 K, as compared in Figure S5 of Supporting Information Note 6. At ***B***-fields above 4 T and *V*_DS_ above 1.2 V, a series of small peaks emerge and the separation between these peaks enlarges with increasing ***B***-fields, as indicated by the black arrows in Figure 3(a). We believe these peaks originate from the splitting Landau levels of FLG. To examine the effect of ***B***-fields on ML-MoS_2_, a zoom-in plot of d*I*/d*V*–*V*_DS_ curves of Figure 3(a) in the low-bias region is shown in Figure 3(b). As can be seen, the measured kink of K points is just broadened as the ***B***-fields increase from 0 to 16 T, as illustrated by the pink-shaded region in Figure 3(b). By contrast, the peak P and the kinks of the Q_1_ and Q_2_ points almost remain unchanged even at high ***B***-fields of 16 T. In general, the energy separation of the two neighboring Landau levels under external ***B***-fields for a parabolic energy band is estimated as Δ*E* = *ℏqB*/m*, where *ℏ* is the reduced Planck constant and *q* and m* are the elemental charge and effective mass of an electron, respectively. Since the effective mass is related to the band curvature, the calculated m* of a five-layer graphene and ML-MoS_2_ follow the relationship *m*_K_^*^(FLG) ≪ *m*_K_^*^(MoS_2_) < *m*_*Q*_1__^*^(MoS_2_) ≈ *m*_*Q*_2__^*^(MoS_2_), which suggests a much smaller energy separation between Landau levels in ML-MoS_2_ than that in FLG (see the detailed calculation in Figure S6 and equations S2–S8 of Supporting Information Note 7). Figure 3(c) plots the calculated Landau fan diagram of five-layer graphene (*K*_*FLG*_) (upper panel) and K and Q valleys of ML-MoS_2_ (lower panel) using the tight binding model. The Landau levels of five-layer graphene exhibit a larger separation with increasing ***B***-fields. On the contrary, the Landau levels of K and Q valleys of ML-MoS_2_ can hardly be distinguished in the ***B***-field range of our experimental measurements. The effect of the ***B***-field is therefore manifested as a broadening of the energy bands, as can be seen in the lower panel of Figure 3(c). These different characteristics of the Landau level in FLG and ML-MoS_2_ under external ***B***-fields are in very good agreement with our experimental observations (see Figure 3(a) and (b)). The evolution of d*I*/d*V*–*V*_DS_ curves at *V*_DS_ values smaller than 1 V under perpendicular magnetic fields further validates the observed kinks originated from the phonon-assisted tunneling related to K, Q_1_, and Q_2_ valleys of ML-MoS_2_, respectively.

## Conclusions

We have systematically investigated the band structures of ML-MoS_2_ using phonon-assisted resonant tunneling in the single-barrier RTD, which is composed of the gate non-tunable and tunable regions. In the gate non-tunable region, the resonant tunneling between the quasi-Fermi level of FLG and the conduction band minima at K, Q_1_, and Q_2_ points of ML-MoS_2_ have been clearly observed in the *I*–*V* measurements, providing a comprehensive snapshot of the band structures of ML-MoS_2_. Furthermore, in the gate tunable region, a gate voltage can make the ML-MoS_2_ more conductive and reduce the contact resistance between the bottom-FLG and ML-MoS_2_. These facilitate a more accurate determination of the band structures of ML-MoS_2_, which show excellent agreement with theoretical calculations. Our findings not only offer a comprehensive physical insight into the band structures of ML-MoS_2_ using gate controlled RTS but also provide an accurate and effective experimental tool to probe the electronic band structures of semiconducting TMDCs.

## Experimental Section

The *h*-BN for capping layer cover (10–20 nm) and barrier (∼3 nm) and few-layer graphene were mechanically exfoliated on a SiO_2_ (300 nm)/Si (n+-doped) substrate, and the monolayer MoS_2_ is exfoliated on PDMS (DGL-17mil-X4, Gel-Pak) from a flux-grown bulk crystal bought from hq-graphene and then transferred on top of the bottom FLG. The capping layer *h*-BN, top FLG, and barrier *h*-BN were stacked in series using the pick-up method^44^ and finally dropped down on top of MoS_2_ to finalize the fabrication of the single-barrier resonant tunneling diode as demonstrated in the schematic drawing in Figure 1(a). After pick-up, we annealed the device at 130 °C for ∼30 min to release the possible strain introduced in the transfer or pick-up process. The barrier *h*-BN is carefully placed to ensure the effective separation between top and bottom FLG. The Au/Cr (70 nm/10 nm) electrodes are patterned by standard electron beam lithography and thermal evaporation process. The electric measurements were conducted in the PPMS system with a temperature range of 2–360 K and ***B***-field ranging from 0 to 16 T.

## Supporting Information Available

Optical image of an RTD, the categorization of the gate non-tunable and tunable region in our RTD, the reproducible measurements of d*I*/d*V*–*V*_DS_ curves of another RTD, the theoretical calculated conduction band structure and density of state of an ML-MoS_2_, the discussion of the dual-plate capacitor effect, the temperature dependence of d*I/*d*V*–*V*_DS_ curves of RTD, and the comparison of Landau level splitting under ***B***-fields between FLG and ML-MoS_2_ (PDF)
