# Supplementary material for: Probing Electronic Band Structure of Monolayer MoS2 in Gate-Controlled Resonant Tunneling Diodes
Source: ACS Appl Mater Interfaces. 2025 Apr 15;17(17):25915–21. doi: 10.1021/acsami.4c21712 (PMC12051169; doi:10.1021/acsami.4c21712)
Supplement: Supplementary file 1 — am4c21712_si_001.pdf [file am4c21712_si_001.pdf]

Supporting Information

Probing Electronic Band Structure of Monolayer MoS_2 in Gate Controlled Resonant Tunneling Diodes

Chengjie Zhou[1], Hui Li[2], Zhenqiao Huang[1], Chun Yu Wan[1], Zijing Jin[1], Junwei Liu[1] and Jiannong Wang[1,*]

[1]Department of Physics, the Hong Kong University of Science and Technology, Hong Kong, China

[2]National Key Laboratory of Opto-Electronic Information Acquisition and Protection Technology and Institute of Physical Science and Information Technology, Anhui University, Hefei, China

*Corresponding author: phjwang@ust.hk

Note 1. Optical image of the device discussed in the main text.

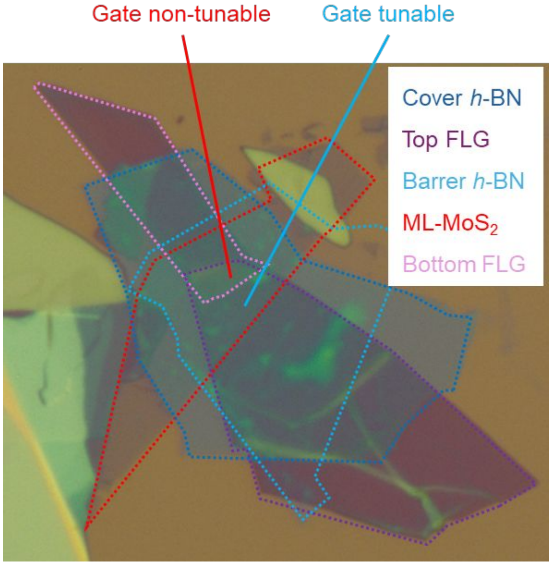

Figure S1 Optical image of the single barrier resonant tunneling diode device measured in the main text. The cover *h*-BN, top few-layer graphene, barrier *h*-BN monolayer MoS_2 and bottom few-layer graphene layers are indicated by the enclosed short dash lines. The gate non-tunable and tunable region are indicated by the red and blue lines, respectively.

Note 2. Categorize the gate non-tunable region and gate tunable region

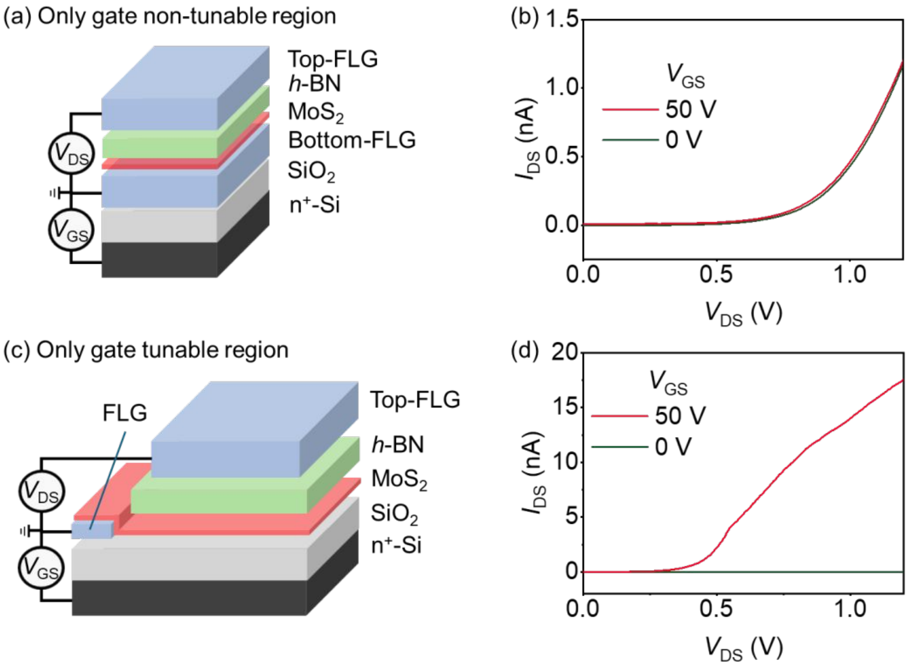

Figure S2 (a) Schematic structure of devices with only gate non-tunable region. (b) The measured I_{DS}-V_{DS} curves of a gate non-tunable region when V_{GS} = 0 V (green curve) or V_{GS} =

50 V (red curve). (c) Schematic structure of devices with only gate tunable region. (d) The measured I_{DS}-V_{DS} curves of a gate tunable region when V_{GS} = 0 V (green curve) or V_{GS} = 50 V (red curve).

To further demonstrate the validity of the separation of two regions of our devices, we have conducted the control experiments, as shown in Figure S2(a-d). Figure S2(a) shows the schematic device structure composed of the stacked Top-FLG/*h*-BN/MoS_2/Bottom-FLG on SiO_2/Si substrate. The top- and bottom-FLG are overlapped, which corresponds to the gate non-tunable region of the devices in Figure 1(a) in the main text. When applying gate voltage from 0 to 50 V, the collected *I-V* curves are unchanged (Figure S2(b)) due to the screening effect of the bottom-FLG. Therefore, the tunneling process is considered to occur between the top- and bottom-FLG with the *h*-BN and MoS_2 layers as a tunneling barrier for the gate non-tunable region. The device structure in Figure S2(c) is similar to that of Figure 2S(a) but without the overlapping of the top- and bottom-FLG, which corresponds to the gate tunable region of the device in Figure 1(a) in the main text. Figure S2(d) shows the measured *I-V* curves of the device in Figure S2(c) at different gate voltages of 0 V and 50 V. As can be seen, the *I-V* curves change significantly with increasing gate voltage due to the transition from an insulating state to a conducting state of the MoS_2 layer at increased gate voltage.

Note 3. Characteristic d*I*/d*V*-V_{DS} curves of another single barrier RTD.

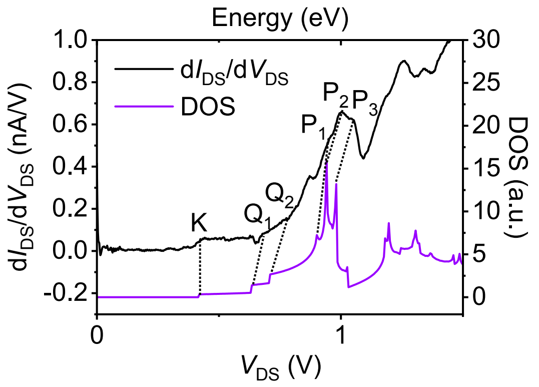

Figure S3 Measured dI_{DS}/dV_{DS}-V_{DS} curve compared with calculated DOS of monolayer MoS_2 for another single barrier RTD.

The three resonant tunneling kinks and one peak between quasi-Fermi level of FLG and K, Q_1, Q_2 and P (P_1, P_2 and P_3) points of ML-MoS_2 are reproducible.

Note 4. Conduction band structure and density of states of a monolayer MoS_2.

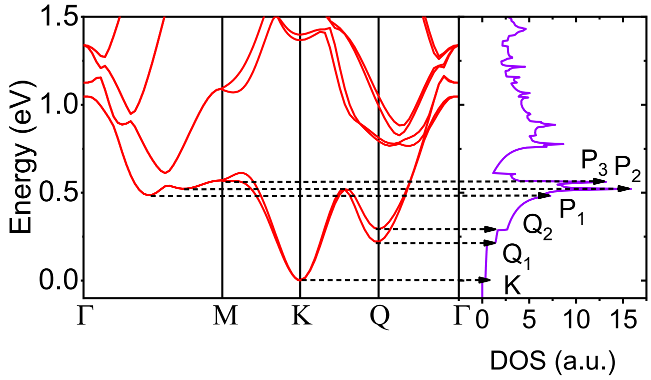

Figure S4 DFT calculated conduction band structure (left panel) and related density of states (DOS) of a monolayer MoS_2 (right panel).

Note 5. Dual plate capacitor effect

The V_{GS} and V_{DS} induced net charge in ML-MoS_2 could be described as,

$$\Delta Q = C_0 V_{GS} - C_1 V_{DS} = \frac{\varepsilon_0 A V_{GS}}{d_0} - \frac{\varepsilon_1 A V_{DS}}{d_1} \qquad \text{(S1)}$$

where ε_0 and ε_1 are dielectric constant of SiO_2 and barrier h-BN, respectively. A is the area of the resonant tunneling region. d_0 (~300 nm) and d_1 (~3 nm) are the thickness of SiO_2 and barrier h-BN, respectively.

Assume $\Delta Q_{threshold}$ is the critical net charge for the alignment between the quasi-Fermi level of bottom-FLG and the conduction band minima in ML-MoS_2. When $\Delta Q > \Delta Q_{threshold}$, the electrons could inject from the bottom-FLG into the ML-MoS_2 to make the ML-MoS_2 to be conductive. Otherwise, the ML-MoS_2 would be insulating.

However, ΔQ is influenced by both the top bias V_{DS} between the top-FLG and ML-MoS_2 and bottom gate voltage V_{GS} between the bottom-FLG and ML-MoS_2. According to Equation (S1), the positive V_{GS} would enlarge the ΔQ, while the positive V_{DS} would reduce the ΔQ. Since $d_1 \ll d_0$ and $\varepsilon_1 \sim \varepsilon_0$, the modulation effect of V_{DS} on ΔQ is greater than that of V_{GS}. Thus, the ML-MoS_2 would be tuned to be insulating again as the V_{DS} increases from 0 V to 1.5 V even at a high V_{GS} of ~80 V, which makes the measured dI/dV-V_{DS} curves to be converged at high V_{DS} > 1.2 V, as shown in Figure 2(a).

Note 6. Temperature dependence of d*I*/d*V*-V_{DS} curves

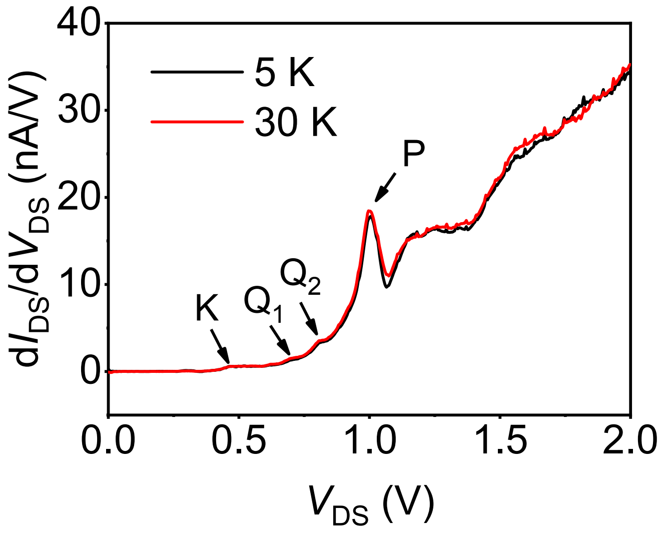

Figure S5 Measured dI/dV-V_{DS} curves at 5 K and 30 K.

As the n^+-doped Si substrates become non-conducting below 20 K, the gate-controlled dI/dV-V_{DS} curves of the RTD could be only investigated at high temperature above 20 K. However, the measured dI/dV-V_{DS} curves at two different temperatures at 5 K (black curve) and 30 K (blue curve) are almost the same except for a slightly broadening of these three peaks of K, Q, and M, as shown in Figure S5 above. Therefore, we conducted the gate-controlled RTS measurements at 30 K and magneto-RTS measurements at 5 K.

Note 7. Landau levels of monolayer MoS_2 and five-layer graphene

The band structures (E-k relation) in the vicinity of K point are assumed to be parabolic following the relationship of

$$E = \frac{\hbar^2 k^2}{2m^*} \tag{S2}$$

where $\hbar$ is the reduced Planck constant, m^* is the effective mass of an electron near the K point and k is momentum. The density-functional theory (DFT) calculated E-k relation of monolayer MoS_2 and five-layer FLG are shown below in Figure S6

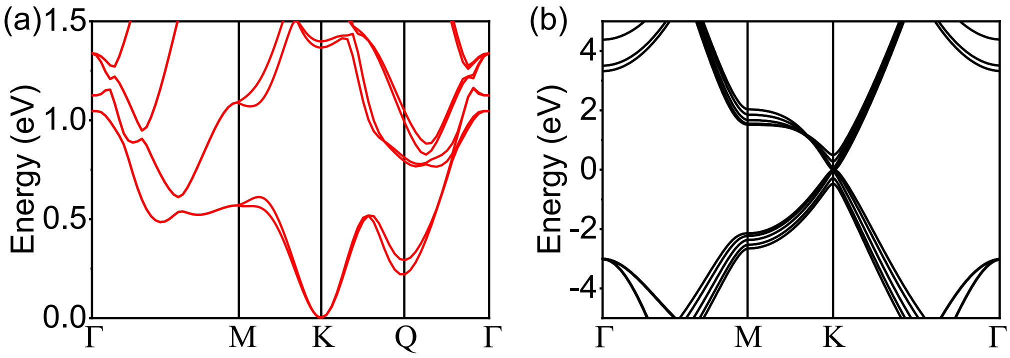

Figure S6 DFT calculated *E*-*k* relation of (a) Monolayer MoS_2 (b) Five-layer FLG.

The effective mass could be deduced from the density-functional theory calculated *E*-*k* relation with,

$$\frac{d^2E}{dk^2} = \frac{\hbar^2}{m^*} \tag{S3}$$

Then the energy difference between two neighboring Landau levels could be estimated as

$$\Delta E = \hbar\omega_c = \frac{\hbar qB}{m^*} \tag{S4}$$

which is the function of the effective mass (m^*) of an electron and the external ***B***-fields. Therefore, at ***B*** = 4 T the estimated ΔE of monolayer MoS_2 and five-layer graphene is calculated to be,

Five-layer FLG at K point:

$$\Delta E = \frac{\hbar qB}{m^*} = \frac{1350\times10^{-12}\ (eVm^2)\times1\ e\times B\left(\frac{Vs}{m^2}\right)}{6.582\times10^{-16}\ (eVs)} \sim 82.0\ meV \tag{S5}$$

Monolayer MoS_2 at K point:

$$\Delta E = \frac{\hbar qB}{m^*} \sim 1.08\ meV \tag{S6}$$

Monolayer MoS_2 at Q_1 and Q_2 point:

$$\Delta E = \frac{\hbar qB}{m^*} \sim 0.76\ meV \tag{S7}$$

Monolayer MoS_2 at M(P_3) point:

$$\Delta E = \frac{\hbar qB}{m^*} \sim 0.1\ meV \tag{S8}$$
